# Pediatric eosinophilic esophagitis: a Belgian single-center retrospective analysis reveals real-life difficulties in diagnosis and treatment

**DOI:** 10.3389/falgy.2024.1478380

**Published:** 2024-11-18

**Authors:** Toon Dominicus, Lisa Nuyttens, Ilse Hoffman, Dominique M. A. Bullens

**Affiliations:** ^1^ Clinical Division of Pediatrics, University Hospitals Leuven, Leuven, Belgium; ^2^ Allergy and Immunology Research Group, Department of Microbiology, Immunology and Transplantation, KU Leuven, Leuven, Belgium; ^3^ Pediatric Gastroenterology, Hepatology and Nutrition, University Hospitals Leuven, Leuven, Belgium

**Keywords:** eosinophilic esophagitis, pediatric, challenges, atopy, endoscopy

## Abstract

**Introduction:**

Eosinophilic esophagitis (EoE) is a chronic immune-mediated disorder characterized by eosinophilic infiltration of the esophageal mucosa.

**Methods:**

This study aimed to provide insights into the clinical characteristics, diagnostic evaluation, treatment modalities, and outcomes of EoE in a pediatric population through a retrospective analysis of 79 patients followed in a single tertiary referral center between 2014 and 2020.

**Results:**

As expected, a higher male prevalence was observed. Median age at diagnosis was 8.9 years, aligning with the typical presentation in childhood, emphasizing the need for early recognition. Clinical presentation varied, with vomiting, dysphagia, and abdominal pain being the most frequently reported symptoms. IgE-sensitization, food allergy and atopy were highly prevalent, with cow's milk, wheat, egg, soy, and peanuts being the most common allergens. Endoscopy results mostly revealed macroscopic abnormalities with linear furrows and microabscesses/white plaques being the most common features although a significant proportion of initial endoscopies (14/79) showed no macroscopic abnormalities, highlighting the importance of esophageal biopsies. Proton pump inhibitors (PPIs) were commonly used as a first-line treatment, with most patients receiving PPI therapy. Other treatment modalities, such as oral budesonide and exclusion diets either single or in combination, were also used. Remission was achieved in 69/79 or 87% patients, with different treatment regimens contributing to successful outcomes but subject to relapse upon time.

**Discussion:**

This study provides valuable insights into the clinical characteristics, diagnostic evaluation, treatment modalities, and outcomes of EoE in the pediatric population. It underscores the importance of early recognition, accurate diagnosis, and regular follow-up to effectively manage this chronic immune-mediated disorder but also demonstrates its complexity in real-life clinical setting.

## 1 Introduction

Eosinophilic esophagitis (EoE) is a chronic type 2 immune-mediated disorder of the esophagus, characterized by the infiltration of eosinophils into the esophageal mucosa (1). It is a disease that affects both adults and children, with a higher incidence in males than females (2, 3). In recent years, there has been a growing interest in the pathophysiology, clinical presentation, diagnosis, and management of EoE.

EoE is considered a rare disease, but its incidence has been steadily increasing over the past few decades. It is estimated to affect 1 in 2,000 individuals, with a higher prevalence in developed countries (4–7). The disease can manifest with a range of symptoms. Dysphagia and food impaction are more prevalent in older individuals, while vomiting and failure to thrive are commonly observed in younger children. Abdominal pain appears consistent across age groups (8). Symptoms may be subtle, including various coping mechanisms and eating behaviors which can be challenging to recognize in daily practice (9). Recognizing these symptoms is vital for identifying and managing EoE effectively.

The diagnosis of EoE requires a combination of clinical, endoscopic, and histologic criteria. The current diagnostic criteria require the presence of symptoms of esophageal dysfunction, endoscopic evidence of esophageal inflammation, and histologic evidence of eosinophilic infiltration of the esophageal mucosa (10).

The management of EoE is multifaceted and requires a multidisciplinary approach. Treatment options include dietary modification, pharmacologic therapy, and endoscopic intervention. The goal of treatment is to achieve symptom relief, improve quality of life, and prevent complications such as strictures and esophageal fibrosis (11).

In this paper, we present a retrospective analysis of 79 pediatric patients with a clinical and histological diagnosis of EoE to provide insight into the clinical characteristics of the disease in our population. We focus on the presenting symptoms, diagnostic evaluation, treatment modalities, and outcomes of these patients.

## 2 Materials and methods

This was a single-center retrospective study conducted at University Hospitals Leuven, a pediatric tertiary referral center in Belgium. We identified all patients diagnosed with EoE between 2014 and 2020 based on histological findings of ≥15 eosinophils per high-power field on esophageal biopsy. Patient demographics, clinical characteristics, endoscopic findings, and treatment history were extracted from electronic medical records. Data analysis was performed using SPSS (version 14, IBM Corp., Armonk, NY). The analysis primarily involved descriptive methods. Continuous variables were evaluated using means, medians, interquartile ranges (IQR) and standard deviations, while categorical variables were characterized using proportions. We employed a Spearman nonparametric correlation analysis, using a two-tailed test, to examine the relationship between follow-up duration and the number of endoscopies performed. Additionally, a Poisson test was employed to assess the difference in diagnostic rates during and outside of the pollen season. Statistical significance was established at 5%.

Inclusion criteria for the study were patients aged ≤ 18 years at diagnosis with histologically confirmed EoE. Patients with incomplete medical records or other causes of esophageal eosinophilia were excluded from the study. All endoscopic procedures were performed by experienced pediatric gastroenterologists using a standardized protocol involving at least 3 level biopsies (proximal-mid-distal esophagus) in duplicate if possible. Treatment of EoE was at the discretion of the treating physician in relation to parents/children preferences and included proton pump inhibitors (PPIs) 1–2 mg/kg/day, topical steroids (oral budesonide 1–2 mg/day), and dietary modifications. Remission was defined as <15 eosinophils per high-power field in all level esophageal biopsies. Specific IgE was measured by CAP (Thermo Fisher Scientific) with sensitization cut-off-titer >0.10 kU/L.

Ethical approval for this study was obtained from the institutional review board. Patient confidentiality was maintained throughout the study, and all data were de-identified before analysis.

## 3 Results

### 3.1 Patient demographics

Our study population comprised 79 pediatric patients diagnosed with EoE, of which 49 were male (62%) and 30 were female (38%). The median age at onset of symptoms was 6.9 years (IQR: 3.9–11.6, range 0–16.7). The median age at diagnosis was 8.9 years (IQR: 4.9–13, range 0.7–17.5). Median delay in diagnosis was 1 year (IQR: 0.1–2.6, range 0–13). Median observation time was 27 months (IQR: 11–24, range 1–113).

### 3.2 Symptoms at diagnosis

**Figure 1:**
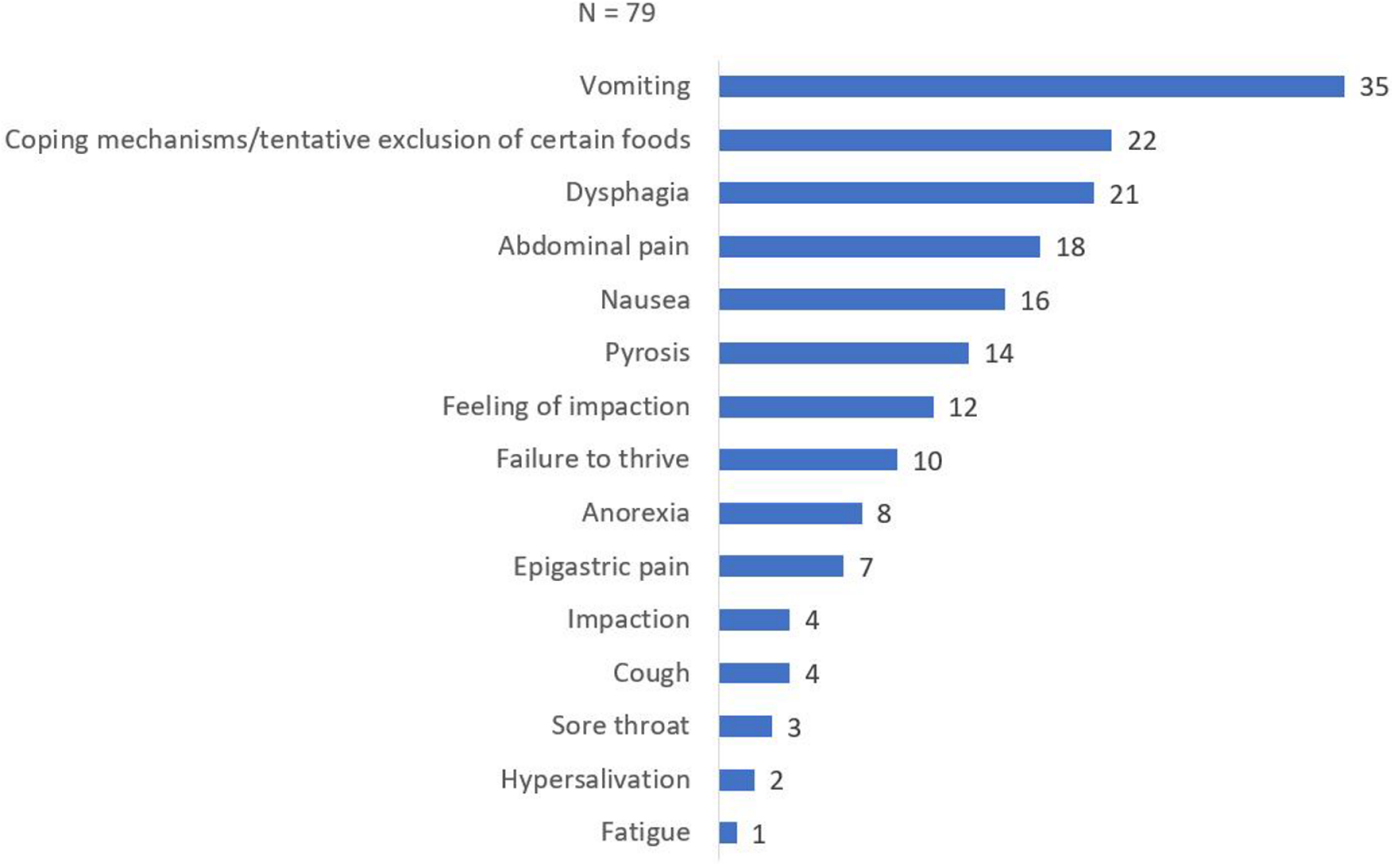
Frequency of symptoms at diagnosis.

Presenting symptoms were documented in all 79 patients and are shown in Figure 1. The most common symptom reported was vomiting, observed in 35 patients (44.3%). Spontaneous adaptation of diet was reported by 22 patients (27.8%), while 21 out of 79 patients (26.6%) presented with dysphagia. Abdominal pain was reported by 18 patients (22.8%), followed by nausea in 16 patients (20.3%). Other presenting symptoms included pyrosis (14/79, 17.7%), feeling of impaction (12/79, 15.2%), failure to thrive (10/79, 12.7%), epigastric pain (7/79, 8.9%), food impaction (4/79, 5.1%), coughing (4/79, 5.1%), sore throat (3/79, 3.8%), hypersalivation (2/79, 2.5%), and fatigue (1/79, 1.3%).

### 3.3 Endoscopy findings

**Figure 2:**
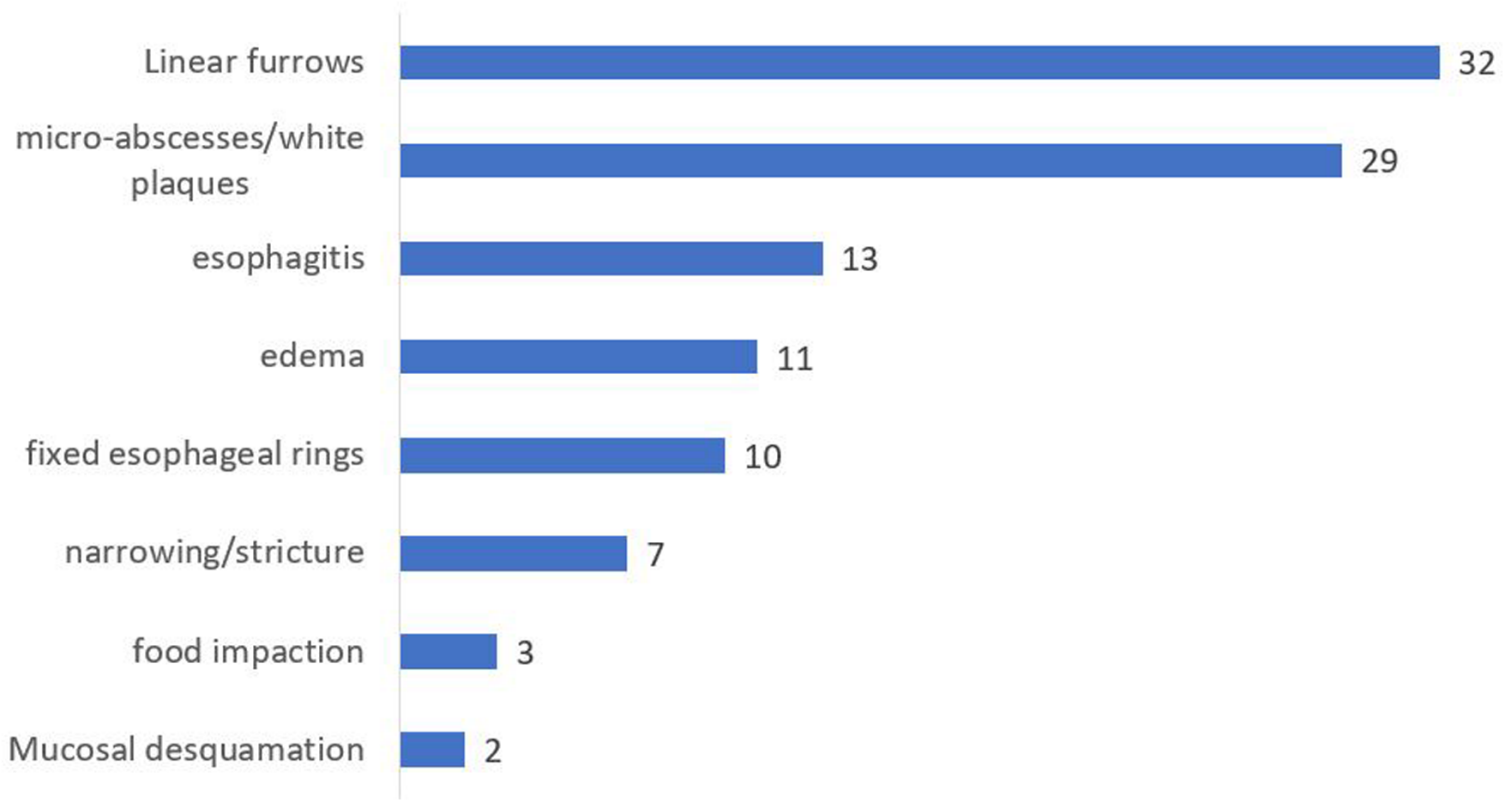
Macroscopic abnormalities noted on initial endoscopy at our center. Interpretation of endoscopy and documentation of abnormalities was at the discretion of the treating pediatric gastro-enterologist.

**Figure 3:**
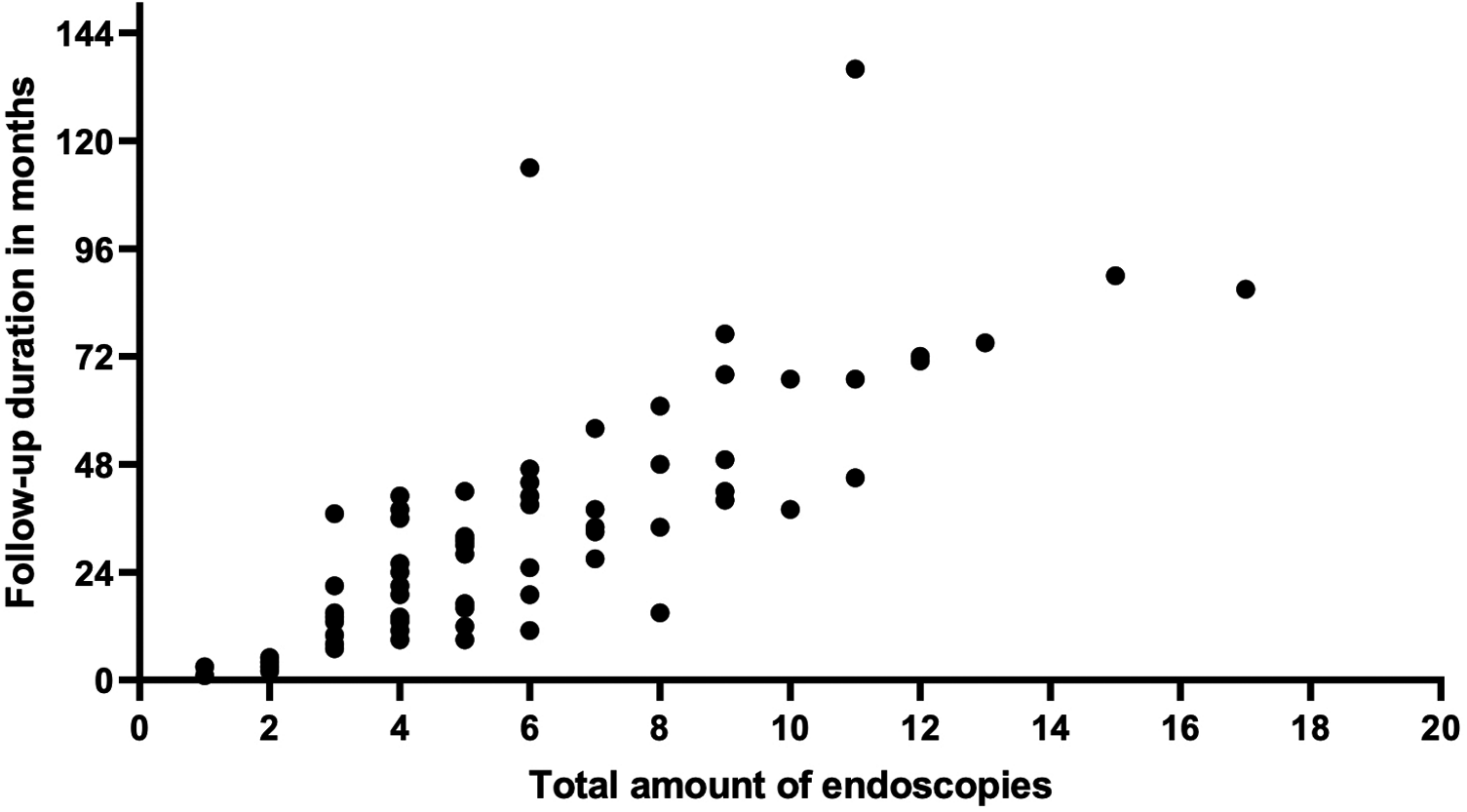
Correlation of number of endoscopies to duration of follow-up in months. Spearman correlation coefficient of r = 0.86 (*p* < 0.001).

All patients showed histological proof of EoE (≥15 eosinophils per high-power field on esophageal biopsy) at baseline and/or during follow-up. Mean number of eosinophils on proximal esophageal biopsy at initial endoscopy was 31.7/HPF (median 25, IQR 25–60, range 0–160) compared to a mean of 44.5/HPF on distal esophageal biopsy (median 30, IQR 19.5–70.5, range 0–200). Number of eosinophils per high power field at first remission and at first relapse are shown in Table 1. Macroscopic appearance of initial endoscopy in our center was normal in 14 out of 79 endoscopies (17.7%). Initial endoscopies were abnormal in 65 out of 79 patients (Figure 2) with the most frequent abnormalities being linear furrows (“train track lesions”) in 32 patients (32/65, 49.2%), followed by microabscesses/white plaques (29/65, 44.6%), esophagitis (13/65, 20%), edema (11/65, 16.9%), fixed esophageal rings (“trachealization”) (10/65, 15.4%), narrowing/stricture (7/65, 10.8%), food impaction (3/65, 4.6%) and mucosal desquamation (2/65, 3.1%). Patients averaged 5.76 endoscopies during the study period (median: 5, range 1–17). There was a positive correlation between duration of follow-up and number of endoscopies performed, indicated by a Spearman correlation coefficient of r = 0.86 (*p* < 0.001) (Figure 3). Out of the 4 patients presenting with food impaction, one patient managed to swallow the impacted bolus before endoscopy was performed, explaining the discrepancy in symptoms at diagnosis and endoscopy findings.

**Table 1:** Number of eosinophils per high power field on biopsy at the proximal and distal esophagus at three different time points (first endoscopy, at time of first remission and at time of first relapse after remission).

	Location	Average	Median	Range	IQR
First endoscopy	Proximal	31.7	25	0–160	25–60
	Distal	44.5	30	0–200	19.5–70.5
Endoscopy at first remission	Proximal	0.99	0	0–16	0–0
	Distal	1.32	0	0–13	0–0
Endoscopy at first relapse	Proximal	25.16	25	0–210	13.5–75
	Distal	38.67	25	0–160	13–54

Upper gastrointestinal series (UGI) were performed in 38 out of 79 patients during the study period. 10 UGI's were abnormal (10/38, 26.3%) with sliding hernia being the most prevalent reported abnormality (6/10, 60%) followed by strictures (3/10, 30%), reflux (2/10, 20%), stasis (1/10, 10%) and abnormal peristalsis (1/10, 10%).

### 3.4 Previous diet and treatment

Information regarding diet and treatment before the first endoscopy was available in all but 1 patient. Seventeen out of 78 patients (21.8%) were reported to be on a diet before the first endoscopy with a diet free of cow's milk being the most common (8/17, 47%), followed by a diet free of nuts (7/17, 41.2%), egg (5/17, 29.4%), peanut (4/17, 23.5%), fish (3/17, 17.6%) and soy (2/17, 11.8%). Various diets excluding different food groups were reported by 4 patients (4/17, 23.5%) with exclusion of at least 5 food groups in 3 patients. All diets were IgE-mediated food-allergy (sensitization and symptoms or specific IgE levels above 95% chance of clinical reactivity for specific food products) related apart from 2 patients who excluded all the food groups mentioned above without concomitant IgE-sensitization (12).

Fourteen out of 78 patients (17.9%) were already receiving treatment before the first endoscopy. All patients already receiving treatment were given proton pump inhibitors (PPIs) with one patient receiving a combination of PPIs and inhaled fluticasone after diagnosis in another hospital before referral.

### 3.5 Atopy before or at diagnosis

Data regarding family history of atopy was available in 73/79 patients with 26 patients showing a positive family history for atopy (26/73, 35.6%). A personal history of atopy since birth was found in 51 out of 79 patients (64.6%) with food-sensitivity being the most prevalent (40/51, 78.4%) followed by allergic rhinoconjunctivitis (19/51, 37.2%), eczema (13/51, 25.5%) and asthma (11/51, 21.6%).

### 3.6 IgE-sensitization at diagnosis

**Figure 4:**
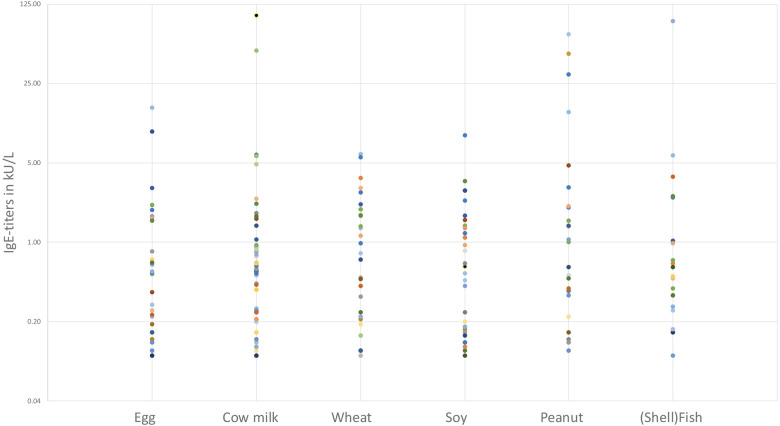
IgE-titer per food group. Each dot represents 1 patient with IgE-titer >0.10 kU/L.

Information regarding IgE-sensitization was available in 78 patients. Fifty-eight patients had a documented, concomitant IgE-sensitization (58/78, 74.3%) with specific allergens identified. Seven out of 58 patients (12.1%) showed a single sensitization (6 for cow's milk, 1 for wheat) whilst 51/58 (87.9%) had multiple sensitizations. The most prevalent food-based sensitization (Figure 4) was observed for cow milk, affecting 42 out of 58 sensitized patients (72.4%). Wheat sensitization was observed in 32 out of 58 patients (55.2%), followed by egg (30/58, 51.7%), soy (27/58, 46.6%), peanut (26/58, 44.8%), hazelnut (23/58, 39.7%) and (shell)fish (19/58, 32.8%).

Regarding aeroallergens, birch and grass pollen were most common (both 23/58, 39.7%) followed by tree pollen mixture (14/58, 24.1%) and house dust mite (12/58, 20.7%).

Other various IgE-sensitizations were found in 23 out of 58 patients (39.7%) and consisted of over 20 different food groups, animals and aeroallergens, listed in Table 2.

**Table 2:** Amount of patients with a positive result (IgE > 0.10 kU/L) for various specific CAP-tests.

	CAP-test	*N*		
Food allergens	Potato f 35	9	Lentil f 235	2
	Apple f 49	7	Pumpkin f 225	2
	Banana f 92	7	Pineapple f 210	2
	Peach (rPru p3) f 420	6	Pear f 94	2
	Gluten f 79	5	Barley f 6	2
	Pea f 12	5	Bromelain k 202	2
	Carrot f31	4	Quinoa f 347	2
	Tomato f 25	4	Avocado f 96	2
	Onion f 48	4	Celery f 85	2
	Kiwi fruit f 84	4	Bovine serum albumine e 204	1
	Buckwheat f 11	4	Cauliflower f 291	1
	Oat f 7	4	Broccoli f 260	1
	Sesame seed f 10	4	Rye f 5	1
	Green bean f 315	3	Strawberry f 44	1
	Rice f 9	3	Mutton f 88	1
	Chicken f 83	3	Gliadin f98	1
	Beef f 27	3	Chick pea f 309	1
	Corn f 8	3	Cucumber f 224	1
	Mushroom f 212	1	Orange f 33	1
	Mustard f 89	1		
Aero-allergens	Cat dander e1	4		
	Dog dander e 5	3		
	Alternaria alternata m 6	2		
	Alternaria (rAlt a1) m 229	1		
	Cat (recombinant Fel d1) e 94	1		
	Aspergillus fumigatus m 3	1		
	Cladosporium herbarum m 2	1		
	Rabbit epithelium e 82	1		
Non-food allergens	Latex k 82	1		

Out of 30 individual patients with a documented sensitization for either grass-, birch- or mixed tree pollen, 14 were diagnosed in the 8 months from August-March (outside the pollen season in Belgium) and 16 were diagnosed between April and July (pollen season), pointing to an increased diagnosis rate during the pollen season (*p* = 0.0019).

### 3.7 Treatment

After diagnosis, 72 out of 79 patients were treated with PPIs (91.1%) in either monotherapy or a combination of PPI's, oral budesonide and/or an exclusion diet. A substantial number of subjects already received PPI's prior to diagnosis, based on symptoms potentially related to gastro-esophageal reflux disease. In those children the PPI's were not stopped after diagnosis, explaining the high number of combination therapies involving PPI's.

Thirty-nine out of 79 patients started monotherapy with PPIs (49.4%), 2/79 started monotherapy with oral budesonide (2.5%) and 4/79 started an exclusion diet (5.1%). Thirty-four patients were started on a combination of therapies, with 20/79 receiving PPIs plus an exclusion diet (25.3%), 9/79 receiving PPIs and oral budesonide (11.4%). One patient received oral budesonide and an exclusion diet (1/79, 1.3%) and 4/79 received triple therapy with PPI's, oral budesonide, and an exclusion diet (5.1%).

All but 2 patients (77/79, 97.5%) received PPIs at some point during the study period. Of the 2 patients who never received PPIs, one was lost to follow-up, and one had a probable diagnosis of PPI-resistant EoE from another hospital, a new trial of PPIs was never done during the study period. The 2 patients that were started on monotherapy with oral budesonide were taking PPIs at the moment of diagnosis but continued with only oral budesonide after diagnosis.

Regarding therapy adherence, 33 out of 79 patients (41.7%) made changes to their medication, while 30 patients independently adjusted their diets between endoscopies without consulting a physician.

### 3.8 Remission

Complete histological remission was achieved in 69 out of 79 patients (87.3%) during the study period. Of the 10 patients who never achieved remission, 6 were lost to follow-up, 2 achieved remission outside of the study period, 1 patient had comorbidities that made an exclusion diet impossible, and 1 patient had very poor therapy adherence.

Twenty-two patients achieved complete remission with only PPIs (22/79, 27.8%), 20 with PPIs plus an exclusion diet (20/79, 25.3%) and 11 with PPIs plus oral budesonide (11/79, 13.9%).

Five patients achieved remission with a combination of an exclusion diet and oral budesonide (5/79, 6.3%). Monotherapy with oral budesonide achieved remission in 3 patients (3/79, 3.8%) and monotherapy with an exclusion diet in 7 patients (7/79, 8.8%). Triple therapy achieved remission in 18 patients (18/79, 22.7%).

Nineteen patients achieved remission with more than 1 possible treatment. Out of all remissions, 30 patients relapsed during the study period (30/69, 43.4%), either due to non-adherence to treatment (*n* = 11), physician-guided step-down in treatment (*n* = 10) or physician-guided planned challenge with a previously excluded food group to try to reduce dietary restrictions (*n* = 6). Three patients in remission relapsed without changes to their treatment plans. Possible explanations for this include undisclosed changes in diet/treatment, worsening disease control, or children outgrowing their medication dose. Another hypothesis is that EoE may react to new food groups over time.

## 4 Discussion

Eosinophilic esophagitis is an increasingly recognized chronic immune-mediated disorder characterized by eosinophilic infiltration of the esophageal mucosa. Our retrospective analysis of 79 pediatric patients with EoE provides valuable insights into the clinical characteristics, diagnostic evaluation, treatment modalities, and outcomes of this disease in a real-life Belgian population.

The demographic profile of our study population revealed a higher prevalence of EoE in males compared to females, which is consistent with previous studies (2, 3, 8). The median age at symptom onset and diagnosis aligns with the typical presentation of EoE in childhood, although the disease can manifest at any age. We observed a median diagnostic delay of 1 year, which is consistent with previous research and highlights the need for increased awareness and early recognition of EoE to minimize the impact of the disease on patients' quality of life (8). We examined the medical files of patients with over a year of diagnostic delay to identify contributing factors. Delays were either due to subtle symptoms (*n* = 10), misattribution of symptoms to other conditions (esophageal atresia, epilepsy, autism, psychomotor retardation) (*n* = 7), delayed physician visits (*n* = 4), endoscopies without biopsies after food impaction (*n* = 3), and coping mechanisms masking signs (*n* = 2).

IgE-sensitization was a prevalent finding in our study, with most patients demonstrating concomitant sensitization to specific allergens. Cow's milk was the most common sensitization, followed by wheat, egg, soy, and peanuts. For several of these food products children were known with symptoms related to IgE-mediated food allergy, for which a dietary restriction was already necessary before EoE diagnosis. These findings emphasize the role of allergens in triggering and perpetuating the inflammatory response in EoE. The association between EoE and atopy, as evidenced by personal and family history, further supports the involvement of an underlying atopic diathesis in the development of EoE (1, 13, 14). The high rate of IgE-sensitization was anticipated by the authors, as the investigators' clinical experience at the pediatric gastroenterology clinic had shown that most EoE patients were sensitized to various allergens. This observation served as the primary motivation for reviewing this specific information in the medical files. It is important to note that not all IgE-titers found were clinically relevant, as some titers were elevated without corresponding clinical reactions to these foods documented in the medical files. Nevertheless, these results were sometimes used to initiate IgE-based elimination diets.

Diagnosis was made outside the pollen season on a rate of 1.75 cases per months whereas a rate of 4 cases a month was observed during the pollen season, suggesting increased diagnostic rates during the pollen season, however our study was not adequately powered to prove this observation.

The diverse clinical presentation of EoE was evident in our study, with as previously suggested vomiting, dysphagia, and abdominal pain being the most frequently reported symptoms (15–19). However, other symptoms such as coping mechanisms, nausea, pyrosis, and failure to thrive were also observed. This underscores the challenge of recognizing EoE, particularly in younger children who may exhibit subtle symptoms or exhibit coping mechanisms and eating behaviors that are challenging to identify in routine clinical practice. Given the retrospective nature of this study it is possible that these more subtle signs, especially highly variable and personal coping mechanisms during eating, are severely underestimated.

The recently introduced standardized severity scale for EoE provides a uniform method for documenting the severity of the disease, facilitating consistent assessment and comparison across studies and clinical practices (20). Given the retrospective nature of our study and the reliance on existing medical records, not all patient files contained the comprehensive information required to apply this standardized scale, so no conclusion on the distribution of the severity scale in our cohort could be made.

Accurate diagnosis of EoE requires a combination of clinical, endoscopic, and histologic criteria, as outlined in the current diagnostic guidelines (21). Endoscopies in our study revealed macroscopic abnormalities in most cases, with linear furrows and microabscesses/white plaques being the most common findings. These endoscopic features, along with histologic evidence of eosinophilic infiltration, support the diagnosis of EoE. It is important to note that 17.7% of initial endoscopies were macroscopically normal, proving the importance of esophageal biopsies. Narrowing and/or strictures were reported in 10.8% of initial endoscopies, which is high compared to previous research (8). It is possible this finding is attributed to an interpretation bias given the fact that narrowing is a highly subjective finding, and it was not always possible to differentiate between the two when reviewing the endoscopy reports. It however highlights the need to recognize and treat the disease timely and effectively.

The management of EoE necessitates a multidisciplinary approach, considering dietary modifications, pharmacologic therapy, and endoscopic intervention. PPIs are commonly used as a first-line treatment option, with almost all our patients receiving PPI therapy. Other treatment modalities, such as oral budesonide and exclusion diets, were also utilized either as monotherapy or in combination. It is important to note that the choice of treatment was based on individual patient characteristics and at the discretion of the treating physician and the preferences of parents and children.

In the treatment of pediatric EoE, exclusion diets often go beyond the 2-4-6 food elimination diets commonly described in previous literature (22–25). These diets can be more complex and personalized, incorporating various inputs such as patient/parent preference, IgE-titers and clinically relevant allergic reactions to specific foods. While the basic elimination protocols provide a starting point, many patients in our study had tailored dietary plans that considered their unique allergen sensitivities and medical histories to effectively manage their condition. In our study, it was not feasible to categorize each diet as a 2/4/6-food elimination diet or any other standardized protocol because most patients had a tailored diet, based on IgE-titers, clinically relevant allergies and subjective input from patients or their parents. These individualized diets could sometimes add up to 2, 4, or 6 eliminated foods but showed too much variance to be considered as a uniform treatment option. This individualized approach highlights the complexity and variability of dietary management in pediatric EoE and acknowledges the gap between theory and practice (26).

Our study showed that treatment non-adherence was frequent, with 41.7% of patients changing their medication regimen or diet, some with consent of the treating physician or primary care doctor in order to try to reduce dietary restrictions if possible, but a large group of them at their own initiative. The treatment changes primarily involved either discontinuing medication or reintroducing food groups, reflecting the challenges patients encounter in maintaining their prescribed treatment plans. Assessing the direct impact of these modifications on symptom remission or recurrence was challenging, as treatment plans often evolved concurrently. This complexity made it difficult to pinpoint which specific changes, medication adjustments or dietary modifications, homemade or physician-guided, had the most significant influence on symptom management and disease progression.

In terms of treatment outcomes, our study demonstrated that remission was achieved in substantial proportion of patients, with different treatment regimens contributing to successful outcomes. 27.8% of patients were completely PPI-responsive, which is in contrast with previous studies that show higher rates of PPI-responsiveness in children (27, 28). It is possible that this is due to a selection bias where PPI-responsive patients are less likely to be referred to a tertiary center. Relapses occurred in almost half of the patients in remission during follow-up, highlighting the chronic nature of EoE and the need for long-term management and monitoring.

While nearly all patients achieved histological remission in our study, a significant number continued to experience symptoms that were not attributable to active EoE based on histological analyses. These unresolved symptoms could suggest the presence of alternative diagnoses such as gastroesophageal reflux disease or other conditions with overlapping symptoms. It underscores the complexity of managing EoE comprehensively, as symptom resolution does not always align with histological remission alone. Further exploration and differential diagnosis are crucial to accurately address ongoing symptoms and optimize patient care.

## 5 Conclusion

Our retrospective analysis provides valuable insights into the clinical characteristics, diagnostic evaluation, treatment modalities, and outcomes of EoE in a pediatric population in a tertiary center. Our findings highlight the importance of early recognition, accurate diagnosis, and a multidisciplinary approach for effective management of this chronic immune-mediated disorder. Further research is warranted to enhance our understanding of EoE and optimize its diagnosis and treatment strategies.

It is important to acknowledge the limitations of our study, including its retrospective nature, single-center design, and the potential for selection bias. The generalizability of our findings may be limited to the specific population and setting of our study.

## Data Availability

The raw data supporting the conclusions of this article will be made available by the authors, without undue reservation.
